# Advancement and applications of peptide phage display technology in biomedical science

**DOI:** 10.1186/s12929-016-0223-x

**Published:** 2016-01-19

**Authors:** Chien-Hsun Wu, I-Ju Liu, Ruei-Min Lu, Han-Chung Wu

**Affiliations:** Institute of Cellular and Organismic Biology, Academia Sinica, 128 Academia Road, Section 2, Nankang, Taipei 11529 Taiwan

**Keywords:** Phage display, Peptides, Epitope mapping, Antigen mimics, Drug delivery

## Abstract

Combinatorial phage library is a powerful research tool for high-throughput screening of protein interactions. Of all available molecular display techniques, phage display has proven to be the most popular approach. Screening phage-displayed random peptide libraries is an effective means of identifying peptides that can bind target molecules and regulate their function. Phage-displayed peptide libraries can be used for (i) B-cell and T-cell epitope mapping, (ii) selection of bioactive peptides bound to receptors or proteins, disease-specific antigen mimics, peptides bound to non-protein targets, cell-specific peptides, or organ-specific peptides, and (iii) development of peptide-mediated drug delivery systems and other applications. Targeting peptides identified using phage display technology may be useful for basic research and translational medicine. In this review article, we summarize the latest technological advancements in the application of phage-displayed peptide libraries to applied biomedical sciences.

## Background

Phage display is a selection technique in which a peptide or protein is fused with a bacteriophage coat protein and displayed on the surface of a virion. This technology was first described by George P. Smith in 1985, when he demonstrated the display of peptides on filamentous phage by fusing the peptide of interest to gene III of filamentous phage [1]. Phage-displayed random peptide libraries enable functional access to the peptides and provide a physical link between phenotype (the displayed peptide) and genotype (the encoding DNA); these libraries lend themselves to a screening process in which binding clones are separated from nonbinding clones by affinity purification.

**Fig. 1:**
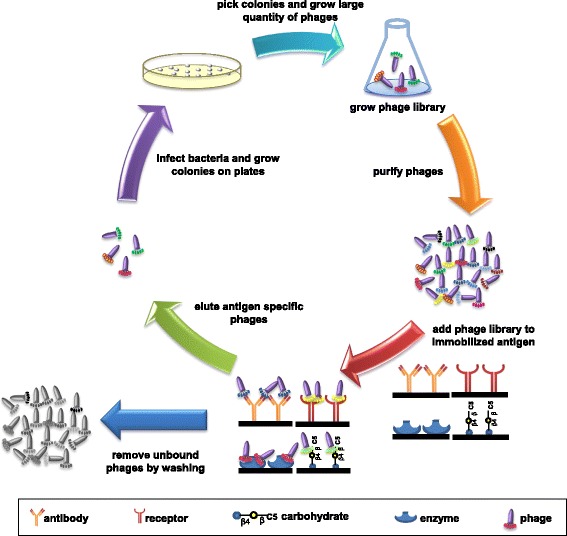
Application of biopanning with phage-displayed random peptide libraries. Random peptide libraries displayed on phages can be used for a number of target candidates, including purified antibodies (B-cell epitopes), receptors (agonists or antagonists), enzymes (enzyme inhibitors), and carbohydrates (antigen-mimetic peptides). After three to five rounds of biopanning, specific individual phage clones are selected and analyzed

Peptides binding to individual targets can be identified by affinity selection (called biopanning). For biopanning, a display library is incubated with an immobilized target, followed by extensive washing to remove nonreacting phages. Binders are usually eluted using acid or high salt and are enriched by amplification in the appropriate host cells. Three to five rounds of biopanning are usually performed in order to obtain targets that bind with high affinity (Fig. 1). The primary structure of the peptide can then be determined by sequencing the DNA of individual clones. Using this approach, it is easy to identify peptides that bind specifically to target molecules.

Phage-displayed peptide library can be used in B-cell and T-cell epitope mapping, selection of bioactive peptides bound to receptors or proteins, selection of disease-specific antigen mimics, selection of peptides bound to non-protein targets, selection of cell-specific peptides, selection of organ-specific peptides, and development of peptide-mediated drug delivery systems and other applications. Targeting peptides identified using phage-displayed peptide libraries have potential use in basic research and translational medicine. In this review paper, we discuss in detail each of the applications of the phage-displayed technology platform in the biomedical sciences.

## B-cell and T-cell epitope mapping

**Fig. 2:**
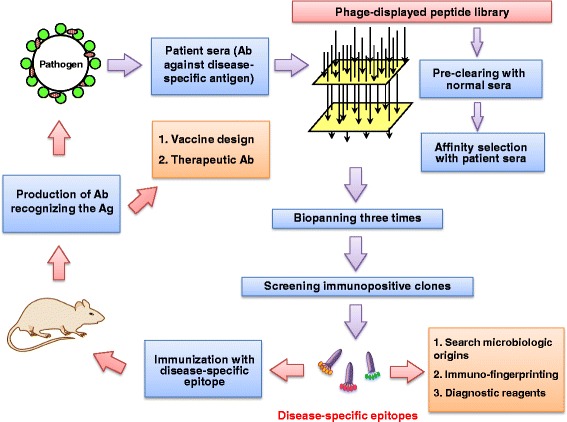
Biopanning selection of phage-displayed peptide libraries using serum samples. The phage-displayed peptide library was precleared using normal human sera and selected with serum antibodies (Abs) from patients with candidate diseases. After three rounds of biopanning, immunopositive phage clones were screened by ELISA. Disease-specific epitopes were further identified and characterized by synthetic peptide binding and competitive-inhibition assays. Phage-displayed disease-specific epitopes can be used to determine microbiological origins, study immunotyping, or provide information for the development of diagnostic reagents and vaccines

Upon encountering antigen, host humoral immunity activates and triggers production of antibodies directed against foreign protein epitopes. Knowledge of these protein epitopes is pivotal in understanding the pathogenesis of pathogen infections and in developing diagnostic reagents, therapeutic antibodies, and effective vaccines. An epitope (known as an antigenic determinant) is recognized by components of the immune system, including antibodies, B cells, and T cells. The epitopes of antigens, which are dependent on their structural properties, can be either linear or conformational [2]. Linear epitopes have some continuous amino acid sequences of antigens, which correspond with their primary structure. In contrast, conformational epitopes contain discontinuous amino acid sequences of antigens, which are based on their protein tertiary structure. There are several experimental methods with which to identify B-cell epitopes, such as Pepscan [3], co-crystallization [4], nuclear magnetic resonance (NMR) [5], computational docking [6], and site-directed mutagenesis [7]. The phage display method provides an economical and rapid approach with which to map B-cell epitopes [8–10]. Previous studies have used various phage-displayed random peptide libraries to identify B-cell epitopes [11–15] or neutralizing epitopes [11, 14, 16] from monoclonal antibodies (mAbs). In addition to mAbs, polyclonal antibodies (e.g. special sera from patients or immunized mice) can also be captured on solid disks or magnetic beads, and then reacted with a comprehensive library of random peptides. Peptides have been selected by biopanning with antibodies from complex sera of patients with various diseases, including severe acute respiratory syndrome (SARS) [17] and infection with human papillomavirus (HPV) [18] and avian influenza viruses (AIV) [19]. Based on information on B-cell epitopes from polyclonal antibodies, certain peptide-based antigens are useful for serological diagnosis, and some are suitable for development of vaccines [17, 20, 21]. The selected disease-specific epitopes may prove to be invaluable for the identification of the etiological agent [17] (Fig. 2).

## Selection of disease-specific antigen mimics

The considerable potential of phage display peptide libraries lies in their capacity to identify some peptide molecules that mimic epitopes (named mimotopes). Mimotopes have fewer similarities to primary amino acids of antigens and always show discontinuous sequences, but they can elicit an identical or highly similar antibody response to that of the native epitope. The development of diagnostic or preventive reagents requires screening phage-displayed peptide libraries for disease-specific epitopes/mimotopes in serum or cerebrospinal fluid samples obtained from patients with viral infections [22, 23], rheumatoid arthritis [24], multiple sclerosis [25], autoimmune thrombocytopenic purpura [26, 27], and neurocysticercosis [28]. Peptides have been selected by biopanning serum samples from patients infected with HPV [18], SARS [17], and AIV [19]. Mimotope-based detection is suitable for broad spectrum antibodies against avian H5N1 influenza virus [29], and has been shown to improve serological detection of SARS [17] and rheumatoid arthritis [30]. Another example is recently identified cholera toxin-binding peptides. Cholera toxin, which is secreted by *Vibrio cholerae*, can enter host cells by binding to GM1, a monosialoganglioside, and would result in acute diarrhea. Biopanning with cholera toxin B (CTB) subunit to select CTB-binding peptides that structurally mimic GM1 could serve as novel agents to block CTB binding on epithelial cells and prevent the ensuing physiological effects of cholera toxin [31].

Mimotopes can also be used to characterize unknown initiating events and provide clues toward disease pathogenesis. Biopanning with antibodies from systemic sclerosis patients was used to screen an immunopositive peptide that had a high degree of similarity to autoantigens, including heterogenous ribonucleoproteins (hnRNP), cytochrome c, fibrillarin, and late protein UL94 of human cytomegalovirus (CMV) [32]. Immunopositive peptide-based affinity purified antibodies from sera of systemic sclerosis patients reacted with a surface component of endothelial cells and induced apoptosis. A dengue anti-NS1 antibody B-cell epitope is cross-reactive to astrocyte elevated gene-1 (AEG-1), a human protein on human endothelial cells that may cause some dengue patients to suffer from hemorrhagic fever (DHF) or dengue shock syndrome (DSS) [33]. Recently, an antigen peptide mimicking alpha-2–Heremans–Schmid glycoprotein, also known as fetuin-A, was identified from serum antibodies of prostate cancer patients [34]. Using this antigen mimic peptide, the authors demonstrated increased serum antibody reactivity to fetuin-A during disease progression in the index patient, and strong serum reactivity in a large cohort of metastatic prostate cancer patients [34]. As mentioned above, study of the disease-specific antigen mimics by phage display helps us to understand the etiology of diseases (Fig. 2).

## Selection of bioactive peptides bound to receptors or proteins

### For receptors

Membrane receptors are pivotal for cell-cell biochemical and electrical signaling in essential physiological functions. Therefore, the pharmaceutical industries tend to focus on developing drugs targeting membrane receptors [35, 36]. Novel receptor ligands discovered using phage-displayed random peptide libraries [37–40] may be either agonists or antagonists (Fig. 1). Two typical examples of agonist peptides selected by phage display are peptides targeting erythropoietin receptor (EpoR) [37] and thrombopoietin receptor (TpoR) [38]. After treatment with Epo mimetic peptide, conformational changes in the extracellular domain of EpoR trigger intracellular signal transduction [37]. This discovery may form the basis for the design of small molecule mimetics of Epo. On the other hand, the selected small peptides targeting TpoR can compete for the binding of the natural ligand, thrombopoietin, and stimulate the proliferation of a TPO-responsive cell line [38]. Antagonists of membrane receptors from phage display have also been reported [41–43]. For example, vascular endothelial growth factor (VEGF) plays an important role in angiogenesis through binding to the kinase domain receptor (KDR/FLK1 or VEGFR2). Peptides as antagonists that block VEGF-mediated angiogenesis have been obtained by phage display [43, 44]. Human CXC ligand 8 (hCXCL8) is a type of chemoattractant that binds to hCXCR1 and hCXCR2, which are involved in inflammation. Inhibition of hCXCL8 binding to hCXCR1 and hCXCR2 by antagonistic peptides has been investigated, and used to develop new therapeutics for treatment of inflammatory diseases [45].

Phage display is also used to isolate receptor-independent peptide modulators, such as G protein-coupled receptors. Some selected peptides from G proteins can interact with the Gαi subunit, leading to an elevated sensitivity of guanine nucleotides to bind to A1 adenosine receptors [46] and to Gβγ subunit in order to enhance the dissociation of G protein heterotrimers in vitro, and activate G protein signaling in intact cells [47].

### For enzyme inhibitors

The pathogenesis of some diseases occurs through the expression of abnormal enzymes, which can serve as potential targets for developing inhibitors as new drugs to block the activities of these enzymes. Phage display has been used to identify the peptide substrate inhibitors that modulate enzyme activities [48, 49] (Fig. 1). Because filamentous phages are resistant to a wide range of proteases, they have been used to identify substrates and generate protease inhibitors [50]. Bahudhanapati et al. identified selective inhibitors of collagenase-1 (metalloproteinase 1, MMP-1) by screening variants of tissue inhibitor of metalloproteinases-2 (TIMP-2) using phage display. TIMP-2 is a broad range inhibitor of matrix metalloproteinases (MMPs) [51]. In addition to protease inhibitors, peptide-based inhibitors against various enzymes, such as human HMG-CoA reductase [52], ubiquitin ligases [53], and tyrosinase [54], have all been identified by phage-displayed random peptide libraries.

### Protein-protein interactions

In cells, protein-protein interactions regulate the mechanisms of several important normal physiological functions. Phage display is a potent and versatile method for studying protein-protein interaction [55–58]. It can be applied to a wide range of protein interaction partners and used in a number of applications, especially in mapping intracellular interactions of the distinct protein domain. Good examples of protein interaction partners include Src homology (SH) 3 domains, which are highly conserved protein interaction modules comprised of 50 to 70 amino acids. SH3 domains are also found in a variety of functionally unrelated proteins. Kärkkäinen et al. generated a library in which all human SH3 domains are expressed on the surface of M13 bacteriophage, thereby enabling analysis of human SH3 domain binding to target proteins of interest, including human immunodeficiency virus-1 Nef, p21-activated kinase (PAK)2, and ADAM15 [59]. Voss et al. also established similar libraries to define the SH3 domain that reacts with the intracellular region of Fas ligand. In addition to the known SH3 domains, the authors also identified a number of additional SH3 domains that may also be associated with FasL [60]. CDBP2 is a cellular adapter protein that contains a GYF domain in its C-terminal fragment. Kofler et al. identified a new conserved motif, PPG (W/F/Y/M/L), in the GYF domain of CDBP2, using phage display. Alignment of these consensus motifs to protein databases, in combination with yeast display or NMR methods, has allowed scientists to rapidly identify novel interaction partners of GYF domain [61, 62].

## Selection of peptides bound to non-protein targets

Many different types of bacteria trigger protective immune responses through cell surface non-protein antigens, such as polysaccharides. Many tumor antigens are carbohydrates. Epitopes mimicking low immunogenic polysaccharides or carbohydrate antigens can be screened and identified by phage-displayed random peptide libraries [63, 64] (Fig. 1). These isolated peptide mimotopes coupled with carrier proteins can be used as potential vaccine candidates to stimulate stronger antibody responses [65, 66].

The phage display technique has also allowed us to identify novel peptides against RNA of interest by screening random peptide libraries [67, 68]. Selective peptides targeting helix 31 of bacterial 16S RNA can be used to inhibit cell-free translation [67]. Bose et al. used phage display to find a selective peptide binding to pre-miR-21, which blocks Dicer processing and decreases miR-21 expression [69].

A few studies have successfully employed peptide phage display to select binders of small molecules, such as fluorophores [70], microcystin-LR [71], and paclitaxel (Taxol) [72]. Recently, Liu et al. performed phage display to screen peptide ligands recognizing an insecticide, imidacloprid, for environmental monitoring in water and soil [73]. The biopanning strategy of phage display can be used to select specific peptides against nanomaterials. These phage-displayed nanomaterial binding peptides are broadly useful in the field of nanotechnology [74]. Whaley et al. showed the power of using combinatorial phage-display libraries to select highly specific peptides that bind to a range of crystalline semiconductor structures, such as GaAs, depending on the nanocrystal orientation [75]. The application of specific peptides has been extended to recognize other substrates of inorganic nanocrystals, including ZnS, CdS, TiO_2_, and SiO_2_, as well as magnetic materials such as Fe_2_O_3_ and Fe_3_O_4_ [76–79]. Peptides derived from phage-displayed libraries can be specifically bound to a conducting polymer for use as a biomaterial to functionalize the surface of conductive polymers, thereby enabling various electronic and biomedical applications [80]. Moreover, the Au-, Ag-, Ti-, Pt-, and Pd-binding peptides were all acquired using the combinatorial phage display peptide library [81–84]. Such metal and semiconductor-targeting peptides have been genetically engineered for use in nano- and biotechnology, particularly in the molecular biomimetics field [84].

## Selection of cell-specific peptides

**Fig. 3:**
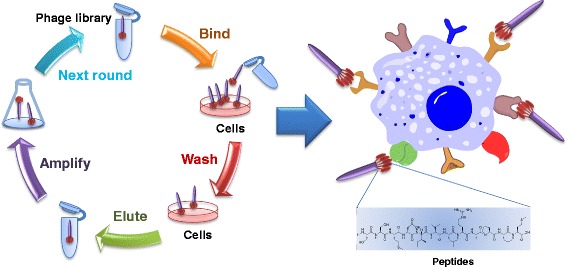
The working principle for screening of cell-specific peptides from a phage display library. A phage display library was incubated with the cells. Unbound phage was removed using washing buffer, and cells bound to phage were eluded and amplified with *E. coli* in broth. Enriched phages were used in the next round of panning. After three to five rounds of selection panning, the positively selected phages were collected and sequenced

Peptide phage display through whole-cell panning offers a high-throughput approach for identifying peptides that bind specifically to a single cell type. Johnston and co-workers were the first to describe the use of peptide-displayed phage to identify peptide binding to several different cell types [85]. The most common screening method is a process called “biopanning”, shown in Fig. 3, in which the binding affinities of the targeted phage clones are enriched. It can be performed in vitro against various types of cells, including cultured cell lines, primary cells isolated from animal models or human patients, or processed cells (fixed cells, activated cells etc.). Within the past ten years, several studies have focused on the in vitro biopanning of phage displayed peptide libraries using various cancer cell types [86–90] to identify cell-specific ligands [91].

Depending on the applications of the ligand, selection can be performed with adherent or fixed cells. The experimental approach can be modified to isolate phages, which bind to the cell surface or peptides, thereby triggering the cellular uptake of the peptides. Peptide-displayed phage libraries are incubated with the cells for a defined period of time. The cells are subsequently washed to remove non-specific and weakly bound phage. In order to reduce the cross-reactivity of the peptide or the phage, blocking agents such as BSA are occasionally used. Removing unbound phage is required to obtain phage clones with strong binding to the desired target, and remove non-specific binding from the background. In general, the washing processes are relatively gentle; however, more stringent washes may increase the affinity of selected phage clones. In some cases, negative selection is performed to avoid the aforementioned problem. In general negative selection is not essential.

Phage bound to the target is recovered using several elution strategies, including the use of acidic buffers, Dithiothreitol, and high ionic strength, which tend to decrease the interaction between the peptide and the target. Most commonly, acidic buffer is sufficient for the elution of target bound phage. However, in the case of strong peptide-target interactions, these elution procedures may only partially break peptide-target interactions, thereby resulting in loss of the high-affinity phage clones. To circumvent this problem, Strukelj and co-workers used a modified method, in which ultrasound was applied during acidic buffer elution to release target-bound phage and enable the selection of high-affinity phage clones [92]. In cases where ligands of a particular target are known and available, competitive elution is the preferred method of isolating the target molecule. This method can specifically elute desired target-bound phage clones while avoiding elution of background-bound phage. Alternatively phage can also be eluted competitively but nonspecifically by using the free target molecule, such as an eluant, or by adding bacterial host directly to the target-bound phage.

Using whole cells instead of purified proteins as target for in vitro biopanning has several advantages. The cellular receptors expressed on live cells can retain their native states (correct protein folding, quaternary structure, expression level, and association with neighboring proteins), and their biological functions and activities. Biopanning with modified protocols can be used for the isolation of peptides that mediate specific cellular functions. For example, selection can be aimed at isolating surface-bound or internalized peptides. Direction elution of phage enables isolation of surface-bound phage. If surface-bound phages are removed by low-pH washes or through treatment with a protease, phage with internalizing characteristics can be isolated. In addition, the use of whole cells for biopanning enables the identification of cell surface molecules with unknown biological functions. This can be used to characterize cell surface profiles and provide information on molecular changes (such as expression level and protein localization) between normal and disease cells.

Although numerous cell-binding peptides have been successfully isolated using in vitro panning against cultured cells, several challenges still remain [91]. In particular, systematic experimental approaches for target identification are lacking [93]. This is a key problem because accurate identification of peptide-targeted molecules is important for basic and clinical research. Conventional receptor identification focuses on membrane protein extraction and affinity purification, followed by mass spectrometric identification of the purified protein. However, the problems associated with this approach arise from the difficulty in maintaining the native interaction between targeting peptide and isolated whole membrane receptor [94]. Furthermore, the binding affinities of targeting peptides are, in general, too low to enable purification by affinity-based methods. Wu and co-workers aimed to overcome the problems outlined above by using biotinylated peptides to directly bind intact cells, and subsequent fixation of ligand-receptor complexes by cross-linking with 3,3′-dithiobis[sulfosuccinimidyl propionate] (DTSSP). After affinity trapping and LC-MS/MS analysis, the unknown target protein on the plasma membrane of the cells could be identified [89]. It is important to note that advances in peptide identification and subsequent receptor identification can lead to the discovery of important cellular targets that were previously unknown. This not only improves our understanding of the molecules expressed in the pathological state, but may also provide useful information on molecules that are not expressed under normal physiological conditions.

## Selection of organ-specific peptides

Organ-specific peptides can be isolated from phage display peptide libraries by performing the selection in a living animal. In vivo phage display technology was first described by Ruoslahti and co-workers in 1996 [95]. They aimed to discover brain vasculature targeting peptides using phage-displayed peptide libraries. Typically, a random peptide phage library is introduced by intravenous injection into the circulation of animal. After a brief circulation time, the animals are sacrificed, and the unbound phage clones are washed off through perfusion of the left ventricle with saline. Based on the half-life of the phage in previous studies, the circulation time of a peptide-displayed phage is generally estimated to be in the 5 to 15 min time range [86, 96, 97]. There is evidence that the displayed exogenous peptide or protein can exhibit circulation half-lives as short as 1.5 min to as long as 4.5 h [98, 99]. Molenaar and co-workers reported that degradation of phage occurs as rapidly as 30 min after injection [98]. A circulation time of over 30 min may be caused by lysosomal degradation of phage after uptake by the target tissue.

**Fig. 4:**
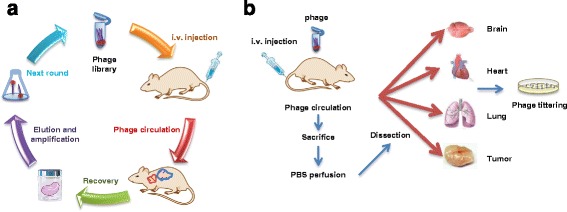
Use of phage display libraries to identify peptides that bind to a specific organ. **a** The in vivo process is based on panning the library of peptides against the target organs. The phage peptide library is injected into the mouse’s tail vein to allow it to circulate throughout the body. After a few minutes in circulation, phage molecules that bind to organs and tissues of interest are isolated. The isolated phage molecules are amplified and subjected to further rounds of selection to enrich organ-specific phage. Finally, DNA from the phage is sequenced to identify the encoded peptide, and the peptide can be used for the development of organ-specific therapeutics and diagnostics. **b** The specificity of selected phage clones can be further confirmed using the phage homing assay. Mice are injected with the selected phage clones through the tail vein. Phage clones are allowed to circulate, and the nonspecific binders are washed out. The organs are subsequently recovered, and the titers of phage in various organs are determined

After removing the unbound phage in circulation, the desired organs are collected and homogenized. The organ-associated phage is recovered from the homogenized tissue. A fraction of the organ lysates are used to infect bacteria for phage amplification and for subsequent rounds of selection in another animal. Another fraction is used for phage titering to measure the amount of recovered phage. After 3–5 rounds of in vivo biopanning, the phage titer recovered from the target tissue should increase [88, 97, 100]. Several peptide motifs are typically identified for a given organ (Fig. 4). After biopanning, the specificity of the isolated peptide needs to be further confirmed. In general, wild type phage without insert or phage with scrambled peptide is used as a negative control. The ability of the isolated peptide to home to the target organ can be confirmed either by comparative analysis using a scrambled peptide, or by competitive studies using a combination of synthetic peptide and selected phage clone with identical peptide sequence. Other approaches, such as immunostaining of homing phage, or fluorescent or radioactive labeling of either phage or synthetic peptide, can be used to directly determine the organ tissue distribution.

This approach has been applied to a number of different organs, including brain, kidney, lungs, liver, uterus, muscle, pancreas, thymus, and mammary gland [101]. Various types of tumor and malignant tissue vasculature have been targeted using phage-displayed random peptide libraries [97, 101–105]. In addition to tumor blood vessels, many tumors induce the growth of new lymphatic vessels and change characteristics during tumor development. Several researchers have used the in vivo phage display technology to map tumor-specific differences in the lymphatic vasculature, and have identified peptides that specifically home to tumor lymphatics [106].

Using in vivo phage display technology to identify specific homing peptides is not without pitfalls. Peptides isolated from animal models using this method may not translate to human because of differences in the vasculature and peptide binding between species. Such limitations can be overcome by performing biopanning in humans. The phage library can be injected into terminal human patients and retrieved from human tissues to identify organ specific peptides [107]. Another study reported the use of phage-display libraries in cancer patients to identify tumor-targeting ligands [108]. In vivo phage display has been shown to be an effective and powerful technique for the isolation of peptides that specifically bind to an organ with high affinity and specificity. However, optimization of these peptides is needed to enhance the clinical applicability of in vivo phage display research.

## Development of peptide-mediated drug delivery systems

**Fig. 5:**
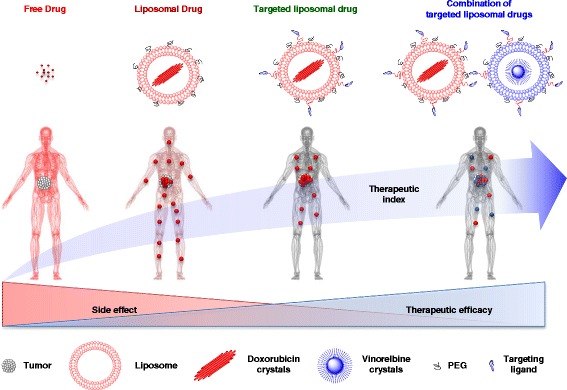
Combination chemotherapy with ligand-targeted delivery of doxorubicin and vinorelbine. Conventional cancer chemotherapy results in the non-specific distribution of toxic therapeutic agents in the human body, which induces adverse side effects. To minimize such side effects, attempts have been made to encapsulate the drug in nanoparticles (e.g., liposomes with encapsulated doxorubicin). Although liposomal doxorubicin has fewer adverse effects than those of free drugs, its therapeutic efficacy is insufficient. The specificity of nanoparticles can be further enhanced through modification with targeting ligand. Some cancer cell types develop drug resistance over the course of drug treatment. The use of targeted nanoparticles to deliver multiple chemotherapeutics (with different mechanisms of action) specifically to cancer cells may simultaneously enhance therapeutic efficacy and reduce undesirable side effects

The delivery of anti-cancer drugs to solid tumors is limited by physical transport barriers within tumors, and such restrictions directly contribute to decreased therapeutic index and the emergence of drug resistance. Drug delivery systems designed to precise spatiotemporal control have demonstrated potential to enhance drug delivery in animal models. Many nanoparticle delivery systems for anticancer drugs have entered the clinic, where they have been shown to exert anticancer effects by improving the pharmacokinetic and pharmacodynamic properties of their associated drugs [109]. Liposomes are the most advanced form of particulate drug carriers. Liposomal systems, like RES-avoiding and long-circulating systems, can confer stable formulation, improved pharmacokinetics, and “passive” targeting of tumor tissue. The newest generation of drug carriers utilizes multicapable nanotechnology-oriented strategies aimed at greater specificity and efficiency. They feature direct molecular targeting of cancer cells via ligand-mediated interactions. Molecular targeted drug delivery can be achieved using targeting liposomes created by linking liposomal drugs to specific ligands. The development of targeting liposomes has been made possible by advances in liposomal systems. These two technologies can in principle be fully integrated, thus combining the specificity of ligands with the drug delivery capabilities of liposomes (Fig. 5).

Peptide-mediated liposomes include three main components: an anticancer drug, a liposome carrier, and targeting ligands (Fig. 5). Remote loading methods, such as the ammonium sulfate method [110] and the pH gradient method [111], can encapsulate weak bases, such as doxorubicin or vinorelbine, into the liposomes with more than 95 % efficiency [89]. Schedule-dependent drugs, such as vinca alkaloids and topotecan, are reasonable candidates for liposomal delivery because of their effectiveness at enhancing the exposure time of cancer cells to therapeutic drug levels. The use of peptide-mediated liposomes also avoids exposing normal tissue to cytotoxic drugs, and prevents adverse side effects. High tumor interstitial fluid pressure (IFP) is a barrier for efficient drug delivery [112, 113]. Increased IFP contributes to decreased transcapillary transport in tumors, which reduces uptake of drugs. This approach sidesteps the problem of high tumor IFP [89, 113, 114] and improves treatment effectiveness, thereby reducing incomplete tumor response, rapid disease relapse, and development of drug resistance due to suboptimal doses, which are often seen when using conventional chemotherapies.

The use of peptides as targeting ligands offers several advantages, including ease of synthesis, structural simplicity, low cost, low immunogenicity, small size, ready diffusion, and simple targeted formulation assembly, as compared to larger biomolecules, such as antibodies. Previous studies showed that using larger biomolecules as targeting ligands may increase the clearance of antibody-modified nanoparticles from the blood [115–117]. This may be due to non-specific binding and uptake of nanoparticles by the RES [116]. Earlier studies with other targeted delivery systems have suggested that targeting moieties increase drug accumulation in tumor tissues [118, 119]. However, targeted nanoparticles have not always caused a significant increase in overall tumor accumulation as compared to the non-targeted drugs. The use of macromolecule-targeting ligands, such as antibodies [120] or transferrin [121], has a negligible impact on tumor accumulation and biodistribution. The differential effects of nanoparticles modified with macromolecules and those modified with small molecules may be due to differences in molecular size, affinity, and penetrability of the targeting ligand [122]. It is possible that targeting moieties with high affinity would be subject to greater internalization and degradation by perivascular tumor cells, thereby limiting their penetration of tumors and reducing their tumor retention [123, 124].

Colorectal cancer is one of the most commonly diagnosed cancers and a leading cause of cancer death worldwide. Traditional chemotherapy only has limited therapeutic efficacy due to non-specific delivery to tumor and non-tumor cells, and the development of drug resistance by cancer cells. Therefore, there is an urgent need to develop more tumor-specific targeted drug delivery systems that can more accurately and effectively deliver the anti-cancer chemotherapeutic drugs to the tumor sites. In a recent study, Wu et al. used phage display technology to identify three peptides that could bind to colorectal cancer cells with high specificity and binding affinity [89]. The authors successfully developed a targeted drug by conjugating these peptides to liposomes. This novel targeted liposomal drug combination specifically delivers chemotherapeutics to tumors, resulting in a much higher dose of drugs being accumulated at the tumor site. This significantly increased the tumor inhibition abilities of these two types of chemotherapeutic drugs, and effectively eliminated cancer without inducing any side effects for 150 days, with no trace of recurrence [89]. Thus, this study demonstrated that drugs can be accurately delivered to tumor sites by combining the liposomal drugs with peptides generated using phage display technology, resulting in a next-generation targeted anti-cancer drug delivery system.

Advances in nanotechnology have facilitated a multidisciplinary approach toward the development of an ideal “smart nanodrug delivery system,” which can be decorated with a targeting ligand for diagnostic or imaging uses, in addition to therapeutic agents of interest (Fig. 5). The availability of such “smart nanodrug delivery systems” in the near future will allow us to detect, diagnose, target, modulate delivery of, and track the progression of therapy both remotely and noninvasively.

## Discussion

Combinatorial phage-displayed random peptide libraries are very valuable tools for studying the interaction between peptides and other substances (or materials). In the past, scientists have been focused on identifying B/T cell epitopes [11–19], disease-specific antigen mimics [22–34], receptor agonists/antagonists [35–45], enzyme inhibitors [48–54] and protein partners [55–62]. In recent years, there is an increasing number of researchers who apply this technique to new areas of studies, such as chemistry [70–72] and materials [74–84] science. Although phage display is a powerful technique, it still has some disadvantages. Choosing suitable phage display libraries (i. e. the number of phage-displayed amino acid residues), using applicable selection condition, ensuring the stability and the quality of phage display libraries and following the appropriate screening protocols are all important factors that could impact the quality and desirability of the ligand peptides generated. If these key parameters can be established, functional target peptides are more likely to be obtained. Furthermore, combination of peptide information from phage display with bioinformatics resources may improve the quality of peptides. Tian et al. [125] and Sandman et al. [126] incorporated genetically-encoded non-natural amino acids into phage-displayed libraries and paved the way for wider chemical diversities. Woiwode et al. [127] also combined a new phage-displayed hybrid system with synthetic chemistry through one-compound-one-clone principle (each compound was encoded by a unique nucleotide sequence inserted in a non-coding region of the phage genome). These new techniques help to further advance the possible applications and potentials of phage display going forward.

Cancer cells, different from normal cells, are often found to overexpress certain antigens. Molecules which can recognize these tumor antigens with high specificity are suitable candidates as potential agents for guiding cancer chemotherapy to target tumor sites. The therapeutic monoclonal antibody, antibody-drug conjugates (ADCs), peptide-drug conjugates (PDCs) and peptide-mediated drug delivery systems have a major impact on the field of cancer therapy. Therapeutic monoclonal antibody based therapies have achieved remarkable clinical success and become one of the most important strategies for treating patients with hematological malignancies and solid tumors. The direct action of antibodies by receptor blockade or agonist activity may have limited therapeutic activity. Antibody-dependent cellular cytotoxicity (ADCC) and complement-dependent cytotoxicity (CDC) have been demonstrated to have a major role in antibody efficacy [128, 129]. Unfortunately, antibody treatment of patients with malignant tumors may not achieve therapeutic effect due to immune suppression, immune escape, complement inhibition etc. ADCs offer the possibility to overcome this issue by enhancing cytotoxicity to cancer cells, thereby reducing undesired side effects. Although ADCs have already been approved as anticancer therapy, there remain several limitations to this type of therapeutics, such as tumor penetration, high manufacturing costs, and challenging conjugation chemistry [130].

PDCs have similar promise as ADCs but they differ in their pharmacological outcomes. Compared to ADCs, PDCs have additional advantages of having smaller molecular mass, higher tissue penetration, enhanced flexibility and well-defined conjugation chemistry, and faster and easier synthesis process when prepared in a homogenous form [130]. Several peptides included RGD motif peptides [131–136], cell penetrating peptides [137–141], and tumor cell specific peptides [142–145] are used for synthesizing PDCs for cancer therapy. After more than decades of research, lots of PDCs have been discovered, and some have been clinically evaluated, although none has yet received regulatory approval. One of the PDCs that has been clinically evaluated and carry most promising is GRN1005. GRN1005 is a LRP-1-targeted peptide-drug conjugate that covalently links paclitaxel to the proprietary 19 amino acid peptide angiopep-2, in a 3:1 ratio. The results from the phase I trial of GRN1005 showed the drug candidate to have acceptable safety profile. GRN1005 is currently undergoing Phase II trials to demonstrate the efficacy, safety, and tolerability of GRN1005 in patients with brain metastases from non-small cell lung cancer (NCT01497665). In addition, GRN1005 in combination with Trastuzumab (Herceptin®) has advanced to Phase II studies as a therapeutic agent for HER2 positive metastatic breast cancer (NCT01480583) [146]. However, one major drawback of PDCs that limits their clinical uses is their stability. PDCs are highly proteolytic unstable, which results in their short half-life in circulation and poor pharmacokinetics. In addition, the relative small molecular weight of PDCs would be rapidly eliminated through renal excretory system.

Neither ADCs nor PDCs allows for high drug payloads on the conjugates molecule. The major challenges facing most ADCs and PDCs are their relatively low capacity for drugs. Hence, more effective payload strategies using different carriers are urgently needed. In addition to ADCs and PDCs, nanoparticles modified with targeting ligand are more effective payload strategies. Nanoparticles as potential vehicle for encapsulating chemotherapeutics can achieve higher drug-loading efficiency. Nanoparticles have been shown to increase the drug-loading capacity by approximately a thousand-fold compared to ADCs and PDCs [89]. Much enhanced drug loading capacity and higher tumor cell specificity render nanoparticles as potentially more promising targeted drugs as a result of their higher efficacy and lower toxicity [89]. ADCs, PDCs and peptide-mediated drug delivery systems have shown considerable promise in improving the delivery of drug to tumor and limiting off-target toxicities simultaneously. These targeting therapeutics represent promising new frontier in cancer treatment.

Despite having the advantages of increased therapeutic efficacy, many challenges still remain for nanoparticles, including potential for off-target effects, ligand stability, immune responses triggering and drugs delivery efficiency in the cytoplasm of target cells. In particular, the rapid clearance of these nanodrugs by the cells of the reticuloendothelial system (RES)/mononuclear phagocyte system (MPS), especially liver and spleen, can lead to increased toxicity to the off-target organs and reduced therapeutic efficacy. However, a recent publication suggested that such off-target effects can be reduced by Intralipid 20 % (a FDA-approved fat emulsion used as parenteral nutrition source), which could decrease the toxicities in liver and spleen and increase the bioavailability of dichloro (_1, 2_-diaminocyclohexane) platinum (II)-loaded and hyaluronic acid polymer-coated nanoparticle (DACHPt/HANP) by possibly inhibiting peritoneal clearance and impairing the phagocytic activity of Kupffer cells [147]. Whether such reduced off target effects can also be observed in liposomal based drug delivery system is worthwhile further investigating.

Drug delivery using antibodies or ligands that bind to specific receptor molecules on tumor target cells allow increased drug accumulation at the target tumor site, however, the actual percentage of drug accumulated at the tumor site was often only a few percent of the total dose administered. Identification of effective ligand-receptor interaction may help to improve the effectiveness of active targeting. In the field of targeted drug delivery, scientists still have a long way to go, but this may change within the next couple of years. Over time, the number of targeted drug-delivery nanoparticles approved by the FDA is expected to increase.

## Conclusions

Although cancer drugs continue to be discovered, most of these drugs have only limited efficacy against cancer, with less than ideal ability to prolong the lives of cancer patients. Small molecule drugs have the advantage of having higher tissue penetration abilities, but they are non-specific to tumors and have a relatively short half-life. Protein drugs are highly tumor-specific; however, they have lower tumor penetration abilities due to their larger molecular sizes. Peptide-mediated drug delivery systems combine the potent small molecule drugs with high specificity of peptides, thus leveraging the benefits of the two therapeutic regimens while reducing their disadvantages. An ideal drug delivery system should achieve a high level of clinical efficacy and minimize the adverse effects. The development of targeting liposomes can improve drug delivery to the target tissue and reduce drug distribution to nontarget tissues, resulting in increased therapeutic activity with minimal side effects. Peptide-mediated liposomes that target tumor cells and vasculature represent a new generation of chemotherapeutic delivery systems. They offer superior pharmacokinetics, controlled biodistribution, greater efficacy, and enhanced safety profiles, and simultaneously improve eradication of disease and reduce common side effects. This has been a long sought-after goal in the development of chemotherapeutic drugs. The modular organization of targeting liposome technology makes it possible to combine peptides with a series of liposomal drugs to yield next-generation targeted agents, such as peptide-mediated targeting liposomes. However, to date, no peptide-drug conjugates or peptide-modified nanoparticles have successfully reached the market. However, certain hurdles must be overcome before peptides can be widely used as targeting moieties, including development of the appropriate ligand for the targeted receptor, understanding the mechanisms of ligand-receptor uptake, disposition, trafficking, and recycling, and compliance with Chemistry, Manufacturing, and Control (CMC) requirements.

## Abbreviations

ADCC: antibody-dependent cellular cytotoxicity
ADCs: antibody-drug conjugates
AEG-1: astrocyte elevated gene-1
AIV: avian influenza viruses
CDC: complement-dependent cytotoxicity
CMC: chemistry, manufacturing, and control
CMV: cytomegalovirus
DHF: hemorrhagic fever
DSS: dengue shock syndrome
DTSSP: 3,3′-dithiobis[sulfosuccinimidyl propionate]
EpoR: erythropoietin receptor
hCXCL8: human CXC ligand 8
hnRNP: heterogenous ribonucleoproteins
HPV: human papillomavirus
IFP: interstitial fluid pressure
mAbs: monoclonal antibodies
MMPs: matrix metalloproteinases
MPS: mononuclear phagocyte system
PAK: p21-activated kinase
PDCs: peptide-drug conjugates
RES: reticuloendothelial system
SARS: severe acute respiratory syndrome
SH: Src homology
TIMP-2: metalloproteinases-2
TpoR: thrombopoietin receptor
VEGF: vascular endothelial growth factor
